# Association between maternal erythrocyte polyunsaturated fatty acid levels during pregnancy and offspring weight status: A birth cohort study

**DOI:** 10.3389/fnut.2022.978679

**Published:** 2022-09-29

**Authors:** Shengchi Wu, Feng Zhao, Yannan He, Tingchao He, Sufang Duan, Gang Feng, Yujing Chen, Xin Wang, Ignatius Man-Yau Szeto, Lizi Lin, Li Cai

**Affiliations:** ^1^Department of Maternal and Child Health, School of Public Health, Sun Yat-sen University, Guangzhou, China; ^2^Institute of Nutrition & Health, Qingdao University, Qingdao, China; ^3^Yili Maternal and Infant Nutrition Institute, Inner Mongolia Yili Industrial Group Co., Ltd., Hohhot, China; ^4^Inner Mongolia Dairy Technology Research Institute Co., Ltd., Hohhot, China; ^5^Nutrition and Health Research Center, National Center of Technology Innovation for Dairy, Hohhot, China; ^6^Department of Occupational and Environmental Health, School of Public Health, Sun Yat-sen University, Guangzhou, China; ^7^Guangdong Provincial Key Laboratory of Food, Nutrition and Health, School of Public Health, Sun Yat-sen University, Guangzhou, China

**Keywords:** polyunsaturated fatty acid, pregnancy, offspring, weight status, birth cohort

## Abstract

**Background:**

The findings of the association between maternal polyunsaturated fatty acid (PUFA) levels during pregnancy and offspring weight status are controversial. Furthermore, few studies have focused on Asian populations or used erythrocyte membranes as biological markers. We aimed to examine the associations between maternal erythrocyte PUFA and offspring weight status within the first 2 years among the Chinese population.

**Materials and methods:**

A total of 607 mother-child pairs were recruited from a birth cohort. Maternal erythrocyte n-3 and n-6 PUFA during pregnancy were measured by gas chromatography, and the ratio of PUFA was calculated. Weight- and body mass index (BMI)-for-age z (WAZ and BAZ) scores were calculated for offspring at 1, 3, 6, 8, 12, 18, and 24 months of age. The risk of overweight and obesity was defined by the WHO criterion. The Generalized Estimating Equation (GEE) model was carried out for repeated anthropometric data within 2 years of age.

**Results:**

Maternal erythrocyte docosapentaenoic acid (DPA, n-3) was inversely associated with offspring BAZ score [tertile 2 vs. tertile 1, β: −0.18 (−0.29, −0.00)]. Higher maternal erythrocyte arachidonic acid (AA) was inversely associated with lower offspring WAZ and BAZ [tertile 3 vs. tertile 1, β: −0.18 (−0.35, −0.02), −0.22 (−0.38, −0.06), respectively]. Furthermore, higher maternal erythrocyte AA [tertile 3 vs. tertile 1, odds ratio [OR]: 0.52 (0.36, 0.75), *p*_*trend*_ < 0.001] and total n-6 PUFA [tertile 3 vs. tertile 1, OR: 0.56 (0.39, 0.81), *p*_*trend*_ = 0.002] were associated with decreased risk of overweight and obesity in offspring. Maternal erythrocyte n-6/n-3 PUFA and AA/eicosapentaenoic acid (EPA) ratios were not associated with offspring weight status.

**Conclusion:**

Maternal erythrocyte PUFA might influence offspring weight status within 2 years of age in the Chinese population. Further Asian studies are still needed.

## Introduction

The increasing prevalence of childhood overweight and obesity remains a serious health concern (1). Public prevention of childhood obesity is urgently needed, and the first 1,000 days of life are a unique window (2). The intrauterine nutritional environment might affect fetal growth and the risk of disease later in life by affecting developmental programming (3), among which polyunsaturated fatty acids (PUFA) are critical nutrients for fetal growth and development (4). It was suggested that the maternal PUFA levels during pregnancy have a long-term effect on offspring weight status.

Several *in vitro* and animal studies have suggested that PUFA could affect fetal adipose tissue accretion. However, the effect may vary by the type and ratio of PUFA (5, 6), especially the composition and ratio of n-3 and n-6 PUFAs. It was suggested that the maternal n-6/n-3 PUFA ratio during pregnancy was more closely associated with fetal metabolic programming (7). Prospective cohort studies also showed conflicting results. The studies from America showed that higher maternal n-3 PUFA was associated with lower total subcutaneous fat mass and lower odds of obesity in children aged 3 years (8). Similarly, another Netherlands study found that lower maternal levels of n-3 PUFA and higher levels of n-6 PUFA were associated with higher total body fat and abdominal fat levels in childhood (9). However, another American study showed that maternal PUFA was not associated with childhood body mass index (BMI) z scores aged 8 years (10). Similarly, results from randomized controlled trials (RCTs) showed inconclusive effects of n-3 PUFA supplement during pregnancy on offspring BMI or body fat percentage (11), because of differences in design, especially the amounts, balance, and type of PUFA given.

Notably, previous studies are almost exclusively from American and European populations, but evidence from Asian populations has been limited. As the Asian population has different genetic variations and dietary patterns when compared with American or European populations (12), more investigation into Asian populations is highly warranted. Moreover, the majority of these studies used plasma as biomarkers or non-objective food frequency questionnaires as exposure measures. Fatty acid composition in erythrocytes could reflect dietary fatty acid intake over the past month or 2 (13), which may be a more stable biomarker than plasma phospholipid fraction. Therefore, we explored the associations between maternal erythrocyte PUFA during pregnancy with offspring weight status within the first 2 years in a Chinese birth cohort.

## Materials and methods

### Study design and participants

Mothers and infants were from a prospective birth cohort study (registration number: NCT03023293), which was carried out at a hospital in Guangzhou, China. We enrolled pregnant women who were aged 20–45 years and at 20–28 weeks of gestation during 2017 and 2018 and then followed up their offspring for 2 years postpartum. Mothers with pre-existing diabetes mellitus, cardiovascular disease, thyroid disease, hematopathy, polycystic ovary syndrome, pregnancy infection, mental disorder, or multiple pregnancies and infants who did not have follow-up data were excluded from the study.

A total of 691 mother-offspring pairs were enrolled. We further excluded women whose erythrocyte PUFA information was missing (*n* = 84). Overall, 607 mother-child pairs were finally included in our analysis. This study was approved by the institutional review boards of Sun Yat-sen University. Informed consent forms were obtained from all participants.

### Maternal erythrocyte fatty acid analysis

Maternal blood samples were collected during 20–28 weeks of gestation. The venous blood samples were collected by professional nurses in the morning after an overnight fast of at least 10 h. The samples were centrifuged at 841 × *g* for 15 min to obtain agglutinated blood cells. Blood cell samples were kept at −80°C until later laboratory analysis.

Laboratory analysis of erythrocyte fatty acid was as follows. First, removed the blood samples from the refrigerator and thawed them. In total, 100 μl of blood cells per sample was dropped on the test paper (Omegabandz Inc., China) and shipped to the laboratory after drying. Second, the tris–HCl buffer was added to the sample. After the red blood cells were hemolyzed, we centrifuged them to obtain the bottom layer of milky red blood cell fragments. And then, the lipid component of erythrocyte fragments was extracted with a chloroformmethanol (2:1, v/v) solvent system containing 10 mg/L of butylated-hydroxytoluene (BHT, Sigma Chemical Co., St. Louis, MO, USA) (14). Third, the methyl esters of the fatty acids from the lipid extract were transesterified with H_2_SO_4_ in methanol (5%, v/v), together with toluene, in sealed tubes at 70°C for 2 h. The methanol layer was transferred to a new test tube, blown by nitrogen, and then dissolved in hexane. Fourth, the derived fatty acid methyl esters were analyzed by using Agilent 7820 Gas Chromatograph (Agilent Corporation, USA) equipped with a 60 m × 0.25 mm × 0.25 μm fused silica-bonded phase column (DB-23, Agilent Corporation, USA) and a flame ionization detector. The column temperature was firstly programmed from 150 to 180°C at a rate of 10°C/min, with an initial hold time of 2 min; then it was increased to 215 at 2.5°C/min and held for 6 min; and finally, it was increased to 230 at 10°C/min and held for another 5 min. Fatty acids were identified by comparison of retention time with standard mixtures of fatty acid methyl ester (Nu-Chek Prep, Inc., Waterville, MN, USA). Quantification of the fatty acid compositions was achieved by the comparison of peak areas with the internal standard (tricosanoic acid, Nu-Chek Prep, Inc., Waterville, MN, USA), which was added to the samples (1 mg of internal standard in 500 mg sample) prior to extraction.

Polyunsaturated fatty acid levels were expressed as a proportion of the total fatty acids. Based on findings from previous studies, selected PUFAs were total n-3 PUFAs, which included α-linolenic acid (ALA, C18:3 n-3), eicosapentaenoic acid (EPA, C20:5 n-3), docosapentaenoic acid (DPA, C22:5 n-3), and docosahexaenoic acid (DHA, C22:6 n-3), and total n-6 PUFA, which included linoleic acid (LA, C18:2 n-6), γ-linolenic acid (GLA, C18:3 n-6), dihomo-gamma-linolenic acid (DGLA, C20:3 n-6), and arachidonic acid (AA, C20:4 n-6). We also calculated the ratio of total n-6/n-3 PUFA and the ratio of AA/EPA.

### Offspring anthropometric measurements

At the age of 1, 3, 6, 8, 12, 18, and 24 months, offspring length and weight without shoes and heavy clothing were measured by the trained nurses. The length was measured to the nearest 0.1 cm by a stadiometer, and weight was measured to the nearest 0.01 kg using an electronic scale. All measurements were performed by trained professionals, and standardized tools were used. BMI was calculated as weight (kg)/height in meters square.

According to the criterion from the World Health Organization Child Growth Standards 2006 (15), offspring weight-for-age z (WAZ) score, length-for-age z (LAZ) score, and BMI-for-age z (BAZ) score were calculated. Furthermore, offspring’s weight status was defined as a normal, possible risk of overweight, or overweight and obesity based on the BAZ score.

### Covariates

Information on maternal age, pre-pregnancy weight and height, educational level (high school or below/junior college/college or above), monthly household income (<4,000/4,000–6,000/6,000–10,000/≥10,000 RMB), and passive smoking during pregnancy were obtained by a face-to-face questionnaire survey in the baseline investigation. Maternal pre-pregnancy BMI was calculated from weight (kg) divided by height squared (m^2^). In addition, all pregnant women were scheduled for a 75-g oral glucose tolerance test between 20 and 28 weeks of gestation. Women were diagnosed with gestational diabetes (GDM) when meeting the criteria of the International Association of Diabetes and Pregnancy Study Groups (16). Offspring sex, birth weight, and other delivery information were obtained from the hospital birth records. Offspring feeding status at 6 months (breastfeeding/formula feeding/mixed feeding) was obtained at the age of 6 months by a structured questionnaire.

### Statistical analysis

All statistical analyses were carried out using SAS statistical software package (version 9.4; SAS Institute Inc., Cary, NC, USA). PUFA levels were divided into three levels based on the tertiles of each PUFA. Continuous variables were reported as the mean ± standard deviation (SD) or median (25th–75th percentile), and categorical variables were expressed as percentages.

Considering the within-subject correlation due to repeated measures, the Generalized Estimating Equation (GEE) (17) was selected to correct for children’s repeated anthropometric measurements at 1, 3, 6, 8, 12, 18, and 24 months of age because the method takes this within-subject correlation into account. After comparing the quasi-likelihood under the independence model criterion (QIC) value, the GEE with six dependent correlation matrices was used to estimate the relationship between the maternal PUFA and offspring WAS score, LAZ score, BAZ score (continuous), and risk of overweight and obesity (ordered categorical) within the first 2 years. The model was adjusted for confounding factors from mothers and infants, which included maternal age, educational level, family income, GDM, pre-pregnancy BMI, passive smoking during pregnancy, infant age, sex, and breastfeeding. *p* < 0.05 was considered significant.

## Results

### Subject characteristics

Table 1 and Supplementary Table 1 show the characteristics of pregnant women and their offsprings. A total of 607 mother-offspring pairs were included in our analyses. The median age of the women was 30.51 years and the median pre-pregnancy BMI was 20.0 kg/m^2^.

**TABLE 1:** Characteristics of pregnant women and offspring (*n* = 607)^a^.

Characteristic	*n* (%) or median (25th–75th percentile)
**Maternal characteristics**	
Age, median (year)	30.51 (26.96, 33.74)
Pre-pregnancy body mass index (kg/m^2^)	20.00 (18.44, 22.19)
Education, *n* (%)	
High school or below	229 (33.78)
Junior college	219 (32.30)
College or above	230 (33.92)
**Monthly household income *n* (%)**	
<4000 (RMB)	119 (17.76)
4000∼6000 (RMB)	163 (24.33)
6000∼10000 (RMB)	172 (25.67)
≥10000 (RMB)	216 (32.24)
Passive smoking during pregnancy, yes (%)	352 (51.69)
Gestational diabetes, yes (%)	131 (18.96)
**Offspring characteristics**	
Males, yes (%)	349 (50.51)
Birth weight (kg)	3.17 (2.95, 3.45)
Birth length (cm)	50.00 (49.00, 50.00)
**Feeding status at 6 months, *n* (%)**	
Breastfeeding	243 (35.2)
Formula feeding	71 (10.3)
Mixed feeding	351 (50.8)

^a^Values represent the median (the 25th, the 75th percentile) or number of subjects (valid %).

Table 2 shows the maternal erythrocyte PUFA levels in mid-pregnancy. The median maternal erythrocyte total n-3 and n-6 PUFA levels were 9.69 and 36.33%, respectively. Among the individual PUFAs, the content of AA was the highest (17.48%), followed by LA, DHA, DGLA, and DPA (15.32, 7.29, 2.43, and 1.25%, respectively). In addition, the ratios of total n-6/n-3 PUFA and AA/EPA were 3.61, and 18.41, respectively.

**TABLE 2:** Maternal erythrocyte PUFA levels during pregnancy (*n* = 607).

	Percentage by weight of total sum of fatty acids (%)^a^
**Total n-3 PUFA (%)**	9.69 (7.86, 11.52)
ALA (C18:3 n-3)	0.25 (0.18, 0.34)
EPA (C20:5 n-3)	0.89 (0.63, 1.36)
DPA (C22:5 n-3)	1.25 (0.99, 1.53)
DHA (C22:6 n-3)	7.29 (5.41, 9.01)
**Total n-6 PUFA (%)**	36.33 (33.42, 38.12)
LA (C18:2 n-6)	15.32 (13.83, 16.69)
GLA (C18:3 n-6)	0.23 (0.15, 0.31)
DGLA (C20:3 n-6)	2.43 (2.08, 2.77)
AA (C20:4 n-6)	17.48 (14.69, 19.05)
**Ratio**	
Total n-6/n-3 PUFA	3.61 (3.09, 4.20)
AA/EPA	18.41 (11.93, 26.66)

^a^Values represent the median (25th–75th percentile).

PUFA, polyunsaturated fatty acids; ALA, α-linoleic acid; EPA, eicosapentaenoic acid; DPA, docosapentaenoic acid; DHA, docosahexaenoic acid; LA, linoleic acid; GLA, γ-linolenic acid; α-linolenic acid; DGLA, dihomo-gamma-linolenic acid; AA, arachidonic acid.

### Maternal erythrocyte polyunsaturated fatty acid ratios and offspring weight status

Table 3 shows that there was an inverse association between medium-level maternal erythrocyte DPA and offspring BAZ scores [tertile 2 vs. tertile 1, β (95% CI): −0.18 (−0.29, −0.00)]. Higher maternal AA levels were associated with lower offspring WAZ score in the adjusted models [tertile 3 vs. tertile 1, β (95% CI): −0.18 (−0.35, −0.02), *p*_*trend*_ = 0.028]. Moreover, higher maternal erythrocyte AA was associated with lower offspring BAZ score [tertile 3 vs. tertile 1, β (95% CI): −0.22 (−0.38, −0.06), *p*_*trend*_ = 0.006]. We did not find associations between other n-3 PUFA or n-6 PUFA with offspring’s WAS score or BAZ score. In addition, Supplementary Tables 2, 3 show the associations of maternal erythrocyte PUFA with the risk of low birth weight and LAZ score in offspring, respectively.

**TABLE 3:** Association of maternal erythrocyte PUFA during pregnancy with offspring weight status^a,b^.

		Weight for age z score				BMI for age z score		
		
	T1	T2	T3	*P*_trend_	T1	T2	T3	*P*_trend_
						
		β (95% *CI*)	β (95% *CI*)			β (95% *CI*)	β (95% *CI*)	
**Total n-3 PUFA**	*Ref.*	−0.06 (−0.22, 0.09)	−0.04 (−0.20, 0.11)	0.597	*Ref.*	0.00 (−0.15, 0.15)	0.05 (−0.10, 0.20)	0.515
ALA (C18:3 n-3)	*Ref.*	0.07 (−0.07, 0.22)	0.05 (−0.11, 0.21)	0.524	*Ref.*	0.05 (−0.10, 0.19)	−0.03 (−0.19, 0.13)	0.690
EPA (C20:5 n-3)	*Ref.*	0.01 (−0.15, 0.16)	−0.05 (−0.2, 0.11)	0.550	*Ref.*	0.10 (−0.05, 0.25)	0.01 (−0.14, 0.17)	0.842
DPA (C22:5 n-3)	*Ref.*	−0.11 (−0.27, 0.04)	−0.06 (−0.22, 0.10)	0.419	*Ref.*	−**0.15 (**−**0.29, -0.00)**	−0.14 (−0.29, 0.02)	0.071
DHA (C22:6 n-3)	*Ref.*	−0.02 (−0.19, 0.14)	−0.1 (−0.27, 0.07)	0.220	*Ref.*	−0.09 (−0.25, 0.08)	−0.08 (−0.25, 0.1)	0.408
**Total n-6 PUFA**	*Ref.*	−0.10 (−0.25, 0.05)	−0.01 (−0.17, 0.16)	0.930	*Ref.*	−0.11 (−0.26, 0.04)	−0.13 (−0.3, 0.03)	0.104
LA (C18:2 n-6)	*Ref.*	0.12 (−0.03, 0.27)	0.12 (−0.04, 0.27)	0.131	*Ref.*	0.10 (−0.06, 0.25)	0.01 (−0.14, 0.16)	0.858
GLA (C18:3 n-6)	*Ref.*	−0.02 (−0.18, 0.13)	0.00 (−0.16, 0.16)	0.955	*Ref.*	0.020 (−0.14, 0.18)	0.04 (−0.11, 0.20)	0.577
DGLA (C20:3 n-6)	*Ref.*	−0.07 (−0.23, 0.09)	0.03 (−0.12, 0.18)	0.699	*Ref.*	−0.06 (−0.21, 0.10)	0.00 (−0.16, 0.16)	0.986
AA (C20:4 n-6)	*Ref.*	−0.02 (−0.18, 0.14)	−**0.18 (**−**0.35,**−**0.02)**	**0.028**	*Ref.*	−0.01 (−0.17, 0.14)	−**0.22 (**−**0.38,**−**0.06)**	**0.006**

^a^Model was adjusted for pregnancy factors and infant factors, which included maternal age, educational level, family income, gestational diabetes, pre-pregnancy body mass index, passive smoking during pregnancy, infant age and sex, and feeding status at 6 months.

^b^Statistically significant results are in bold (*p* < 0.05).

PUFA, polyunsaturated fatty acids; ALA, α-linoleic acid; EPA, eicosapentaenoic acid; DPA, docosapentaenoic acid; DHA, docosahexaenoic acid; LA, linoleic acid; GLA, γ-linolenic acid; α-linolenic acid; DGLA, dihomo-gamma-linolenic acid; AA, arachidonic acid; T3, tertile 3; T2, tertile 2; T1, tertile 1.

Table 4 shows that higher maternal erythrocyte total n-6 PUFA and AA were associated with decreased risk of overweight and obesity in offspring (tertile 3 vs. tertile 1, OR (95% CI): 0.56 (0.39, 0.81), *p*_*trend*_ = 0.002; tertile 3 vs. tertile 1, OR (95% CI): 0.52 (0.36, 0.75), *p*_*trend*_ < 0.001, respectively). Non-significant associations were observed between other maternal PUFAs with offspring’s risk of overweight and obesity.

**TABLE 4:** Association of maternal erythrocyte PUFA during pregnancy with the risk of overweight and obesity in offspring^a,b^.

	T1	T2	T3	*P*_trend_
			
		OR (95% *CI*)	OR (95% *CI*)	
**Total n-3 PUFA**	*Ref.*	0.78 (0.54, 1.14)	1.06 (0.72, 1.54)	0.755
ALA (C18:3 n-3)	*Ref.*	0.95 (0.66, 1.36)	0.97 (0.66, 1.43)	0.888
EPA (C20:5 n-3)	*Ref.*	0.99 (0.68, 1.44)	1.01 (0.67, 1.53)	0.955
DPA (C22:5 n-3)	*Ref.*	0.76 (0.53, 1.10)	0.75 (0.52, 1.09)	0.119
DHA (C22:6 n-3)	*Ref.*	0.74 (0.49, 1.11)	0.69 (0.45, 1.04)	0.089
**Total n-6 PUFA**	*Ref.*	**0.68 (0.46, 0.99)**	**0.56 (0.39, 0.81)**	**0.002**
LA (C18:2 n-6)	*Ref.*	1.26 (0.87, 1.82)	0.84 (0.57, 1.22)	0.408
GLA (C18:3 n-6)	*Ref.*	1.02 (0.70, 1.48)	1.07 (0.7, 1.65)	0.746
DGLA (C20:3 n-6)	*Ref.*	0.84 (0.58, 1.22)	0.76 (0.51, 1.11)	0.152
AA (C20:4 n-6)	*Ref.*	**0.62 (0.42, 0.91)**	**0.52 (0.36, 0.75)**	**<0.001**

^a^Model was adjusted for pregnancy factors and infant factors, which included maternal age, educational level, family income, gestational diabetes, pre-pregnancy body mass index, passive smoking during pregnancy, infant age and sex, and feeding status at 6 months.

^b^Statistically significant results are in bold (*p* < 0.05).

PUFA, polyunsaturated fatty acids; ALA, α-linoleic acid; EPA, eicosapentaenoic acid; DPA, docosapentaenoic acid; DHA, docosahexaenoic acid; LA, linoleic acid; GLA, γ-linolenic acid; α-linolenic acid; DGLA, dihomo-gamma-linolenic acid; AA, arachidonic acid; T3, tertile 3; T2, tertile 2; T1, tertile 1.

### Maternal erythrocyte polyunsaturated fatty acid ratios and offspring weight status

**FIGURE 1:**
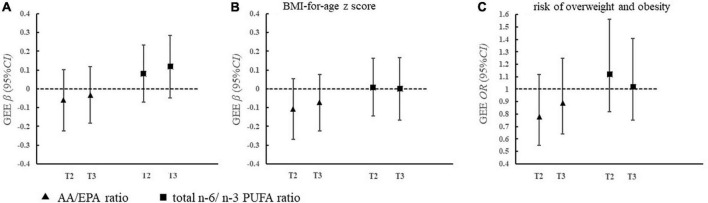
Association of maternal erythrocyte PUFA ratio during pregnancy with offspring weight status **(A–C)**. **(A)**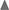 AA/EPA ratio 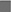 total n-6/n-3 PUFA ratio. **(B)** Model was adjusted for pregnancy factors and infant factors, which included maternal age, educational level, family income, gestational diabetes, pre-pregnancy body mass index, passive smoking during pregnancy, infant age and sex, and feeding status at 6 months. PUFA, polyunsaturated fatty acids; EPA, eicosapentaenoic acid; AA, arachidonic acid; T3, tertile 3; T2, tertile 2; T1, tertile 1.

Figure 1 shows that maternal total n-6/n-3 ratios and AA/EPA ratios were not associated with offspring WAZ score, BAZ score, or the risk of overweight and obesity in the adjusted GEE model.

## Discussion

To the best of our knowledge, this is the first prospective study to explore the association between maternal erythrocyte PUFA during pregnancy with offspring weight status in the Chinese population. We found that higher maternal erythrocyte DPA during pregnancy was associated with lower offspring BAZ score within 2 years old. Higher maternal erythrocyte AA was associated with lower offspring WAZ and BAZ scores. Similarly, maternal erythrocyte AA and total n-6 PUFAs were associated with decreased risk of overweight and obesity.

An appropriate supply of n-3 PUFA during pregnancy has an effect on optimal fetal development (4), but whether the effect can persist into childhood is not clear. DPA is one of the n-3 PUFAs, which can play an independent role. We found a negative association between maternal DPA and offspring BAZ score. Consistent with our results, Vdakovic et al. also observed that higher maternal plasma DPA levels were associated with lower childhood total body fat percentage in the Netherlands (9). The underlying possible mechanisms were as followed. Firstly, it is known that overweight and obesity are characterized by chronic low-grade inflammation, while DPA is the precursor of a large panel of lipid mediators (protectins and resolvins) principally implicated in the pro-resolution of the inflammation, with specific effects (18). Secondly, it has been confirmed that DPA inhibits the process of adipocyte differentiation through the inhibition of the activity of the cyclooxygenase enzymes (19), leading the decreased fat accumulation and expression of inflammatory markers (20). Thus, it is possible that maternal DPA may have a long-lasting effect on offspring growth and development through multiple potential mechanism pathways. It is necessary to further advance the critical window of opportunity for the prevention of lifelong obesity (21).

It is worth mention that we found no association of maternal DHA or EPA with offspring weight or BAZ score. In line with our results, Moon et al. found a non-significant relationship between maternal plasma DHA or EPA and offspring growth (22). Moreover, a recent meta-analysis suggested a null correlation between maternal DHA and EPA supplementation in pregnancy and offspring BMI z score at 0–4 years of age (23). However, in contrast with our results, Donahue et al. found that an enhanced maternal DHA and EPA status was associated with lower childhood adiposity in the American population (8). These discrepancies might be partly explained by differences in the n-3 PUFA levels in various populations. DHA and EPA levels of our population were higher than that of American women in whom differences were found (24) but close to the reference interval for healthy Norwegian pregnant women (25). This indicated that the variation of DHA or EPA in our population might not be large enough, which makes it more difficult to find significant associations. Furthermore, the possible interactions of environmental pollutants with DHA and EPA have complex effects on offspring growth and development (26). Therefore, a larger sample size and diverse research designs may be needed for the Chinese population to further confirm the association of maternal DHA and EPA with offspring WAZ or BAZ scores.

Interestingly, we observed that maternal erythrocyte AA, an n-6 PUFA, was related to lower offspring WAZ score, BAZ score, and decreased risk of overweight and obesity. Consistent with our results (9), Much et al. found that higher maternal AA during pregnancy was associated with lower BMI in offspring at 1 year of age in the German population (27). Similarly, Al-Hinai et al. suggested that Mexican maternal intake of AA during mid-pregnancy was inversely associated with offspring linear growth (25). Nevertheless, the hypothesis that higher maternal AA during pregnancy promotes offspring adiposity was confirmed in some European and American mother-offspring pairs (9, 22). The main reason for the inconsistent results might be that AA played different roles at different levels. Although excess AA can serve as a substrate for the production of many pro-inflammatory mediators, (28) optimal AA during pregnancy is beneficial for fetal brain and immune system development (29). Moreover, AA-derived metabolites also have roles in the resolution of inflammation (30). Compared with the American and European populations, the absolute intake of AA in the Chinese population was lower than the Chinese Recommended Nutrient Intakes (RNIS) and North American recommendations for fat intake. (31). Therefore, it might be possible that appropriate AA during pregnancy promotes offspring growth rather than fat accumulation.

Previous studies have shown that an increase in the n-6/n-3 ratio has accelerated the risk for obesity (32). Over the past few decades, the intake of n-6 PUFA is increased while n-3 PUFA is decreased in the modern Western diet, which has pushed the n-6/n-3 ratios from 1:1 to 15:1 (33). Donahue et al. have found that a higher ratio of cord plasma n-6/n-3 PUFA was associated with a higher risk of obesity in American children (8). However, we did not find the association of maternal n-6/n-3 PUFA ratio or AA/EPA ratio with offspring weight status, consistent with the results of the study conducted in Germany (34). A potential explanation for the discrepant findings was the various levels of n-6/n-3 PUFA ratio in the participants. The ratio of maternal erythrocyte n-6/n-3 PUFAs in our population was 3.61, within the reference interval for pregnant women (25). Studies also suggested that a ratio of n-6/n-3 PUFA lower than 5 could reduce the risk of adverse inflammation (35). The n-6/n-3 PUFA ratio in our study was at an appropriate level and might not cause pathological inflammation in the fetus, which helps us to understand the null association between maternal n-6/n-3 PUFA and offspring weight gain or obesity. Based on the above evidence, maternal n-6 PUFAs, especially AA, during pregnancy may benefit offspring growth and development within 2 years of age in the Chinese population when the ratio of n-6/n-3 PUFAs falls within the appropriate range. However, further studies are still needed to explore whether the association of maternal PUFA ratio with offspring weight status varies by different n-6/n-3 PUFA levels in other populations.

Our study has several limitations. First, although the observational study could not establish an exact causal relationship, our study was a prospective cohort study and we have performed an extensive adjustment for the potential maternal and childhood confounders. Second, due to the difficulty in measuring body composition in young children, we have only measured offspring anthropometric index as the outcome but did not provide information on body composition, which can reflect the fat distribution (36). Nevertheless, the BAZ score of children can still predict obesity in childhood and even in adulthood accurately. Third, the sample size of our study was moderate when compared to previous literature. It was possible that the statistical power for individual fatty acids might be insufficient. Therefore, more future studies are still needed. Finally, the levels of PUFA in pregnant women might change with the prolongation of pregnancy. Our study only measured the maternal PUFA in the second trimester, lacking information about the third trimester. However, the second trimester is a critical period for fetal adipocyte development (37), and the level of PUFA in the third trimester was shown to be similar to those in the second trimester (38). Therefore, it is theoretically appropriate to select the second trimester as the exposure window for our study.

## Conclusion

The maternal erythrocyte DPA, AA, and total n-6 PUFA might influence offspring weight status within 2 years old in the Chinese population. No significant associations were found between maternal n-6/n-3 PUFA or AA/EPA ratio and offspring weight status. Further Asian studies are still needed to assess the effects of maternal PUFA on offspring weight status throughout childhood.

## Data availability statement

The datasets presented in this article are not readily available because they are from an ongoing cohort. Requests to access the datasets should be directed to LC, caili5@mail.sysu.edu.cn.

## Ethics statement

The studies involving human participants were reviewed and approved by the Ethics Committee of the School of Public Health of Sun Yat-sen University. Written informed consent to participate in this study was provided by the participants’ legal guardian/next of kin.

## Author contributions

LC conceived the study. SW performed data curation, statistical analysis, and prepared the manuscript draft. FZ and YH provided guidance on the process of fatty acid detection. YC performed the investigation and carried out quality control. LC, XW, and LL revised the initial manuscript. TH, SD, and IS critically reviewed this manuscript. All authors contributed to the manuscript revision and approved the submitted version.
