# Supplementary material for: Association between maternal erythrocyte polyunsaturated fatty acid levels during pregnancy and offspring weight status: A birth cohort study
Source: Front Nutr. 2022 Sep 29;9:978679. doi: 10.3389/fnut.2022.978679 (PMC9557224; doi:10.3389/fnut.2022.978679)
Supplement: Supplementary file 1 [file Data_Sheet_1.docx]

Supplementary Material

# Supplementary Tables

**1.1 Table S1.** **Weight status of children aged 0-2 years**.

| **Age** | underweight (n%) ^a^ |  | risk of overweight and obesity (n%) ^b^ |
| --- | --- | --- | --- |
| 1 month | 14 (2.55) |  | 56 (10.31) |
| 3 months | 9 (1.70) |  | 93 (17.78) |
| 6 months | 6 (1.17) |  | 85 (16.48) |
| 8 months | 10 (2.00) |  | 50 (10.19) |
| 12 months | 11 (2.14) |  | 53 (10.53) |
| 18 months | 4 (0.88) |  | 54 (12.05) |
| 24 months | 10 (1.84) |  | 54 (10.76) |

^a^ Underweight was defined as weight for age z score <-2.

^b^ Risk of overweight was defined as BMI for age z score>1.

1.2 Table S2 Association of maternal erythrocyte PUFA during pregnancy with the risk of low birth weight in offspring

|  |  | T1 |  | T2 |  |  | T3 |  |
| --- | --- | --- | --- | --- | --- | --- | --- | --- |
|  |  |  |  | *OR* (95% *CI)* | *P* |  | *OR* (95% *CI)* | *P* |
| **Total n-3 PUFA** |  | Ref. |  | 1.19 (0.63 ,2.23) | 0.592 |  | 1.48 (0.81 ,2.70) | 0.207 |
| ALA(C18:3n3) |  | Ref. |  | 1.01 (0.55 ,1.83) | 0.984 |  | 1.01 (0.56 ,1.85) | 0.964 |
| EPA(C20:5n3) |  | Ref. |  | 0.90 (0.48 ,1.69) | 0.751 |  | 1.54 (0.86 ,2.75) | 0.146 |
| DPA(C22:5n3) |  | Ref. |  | 1.04 (0.59 ,1.86) | 0.890 |  | 0.93 (0.50 ,1.71) | 0.803 |
| DHA(C22:6n3) |  | Ref. |  | 1.30 (0.71 ,2.40) | 0.393 |  | 1.16 (0.62 ,2.19) | 0.644 |
| **Total n-6 PUFA** |  | Ref. |  | 0.80 (0.45 ,1.45) | 0.465 |  | 0.79 (0.44 ,1.44) | 0.448 |
| LA(C18:2n6) |  | Ref. |  | 1.41 (0.79 ,2.50) | 0.243 |  | 0.68 (0.36 ,1.31) | 0.247 |
| GLA(C18:3n6) |  | Ref. |  | 1.18 (0.63 ,2.19) | 0.610 |  | 1.64 (0.89 ,3.00) | 0.112 |
| DGLA(C20:3n6) |  | Ref. |  | 2.11 (1.14 ,3.91) | 0.018 |  | 1.28 (0.66 ,2.49) | 0.462 |
| AA(C20:4n6) |  | Ref. |  | 1.08 (0.60 ,1.94) | 0.804 |  | 0.95 (0.51 ,1.76) | 0.864 |
| **Ratio** |  |  |  |  |  |  |  |  |
| Total n-6/n-3 |  | Ref. |  | 0.89 (0.50 ,1.58) | 0.687 |  | 0.64 (0.34 ,1.22) | 0.178 |
| AA/EPA |  | Ref. |  | 0.62 (0.35 ,1.12) | 0.116 |  | 0.63 (0.35 ,1.14) | 0.128 |

*^a^* low birth weight was defined as birth weight <2500g.

*^b^* model was adjusted for maternal age, educational level, family income, gestational diabetes, pre-pregnancy body mass index, passive smoking during pregnancy, infant age.

*^c^* the small number of low birth weight cases (n=19) might lead to low statistical power for the associations between maternal PUFA and low birth weight.

**1.3 Table S3. Association of maternal erythrocyte PUFA during pregnancy with offspring length for age z sore.**

|  |  | T1 |  | T2 |  |  | T3 |  |  | *P _trend_* |
| --- | --- | --- | --- | --- | --- | --- | --- | --- | --- | --- |
|  |  |  |  | *β* (95% *CI)* | *P* |  | *β* (95% *CI)* | *P* |  |  |
| **Total n-3 PUFA** |  | Ref. |  | -0.13(-0.31, 0.05) | 0.145 |  | -0.13(-0.31, 0.05) | 0.168 |  | 0.173 |
| ALA(C18:3n3) |  | Ref. |  | 0.05(-0.12, 0.22) | 0.572 |  | 0.11(-0.07, 0.29) | 0.219 |  | 0.219 |
| EPA(C20:5n3) |  | Ref. |  | -0.10(-0.27, 0.08) | 0.280 |  | -0.06(-0.24, 0.12) | 0.528 |  | 0.520 |
| DPA(C22:5n3) |  | Ref. |  | -0.04(-0.22, 0.13) | 0.629 |  | 0.04(-0.15, 0.22) | 0.695 |  | 0.723 |
| DHA(C22:6n3) |  | Ref. |  | 0.02(-0.17, 0.21) | 0.839 |  | -0.14(-0.32, 0.05) | 0.142 |  | 0.119 |
| **Total n-6 PUFA** |  | Ref. |  | -0.06(-0.24, 0.12) | 0.501 |  | 0.13(-0.05, 0.31) | 0.157 |  | 0.158 |
| LA(C18:2n6) |  | Ref. |  | 0.08(-0.09, 0.26) | 0.363 |  | 0.22(0.06, 0.39) | 0.009 |  | 0.010 |
| GLA(C18:3n6) |  | Ref. |  | -0.06(-0.25, 0.13) | 0.531 |  | -0.05(-0.23, 0.13) | 0.554 |  | 0.558 |
| DGLA(C20:3n6) |  | Ref. |  | -0.04(-0.21, 0.14) | 0.680 |  | 0.03(-0.15, 0.20) | 0.746 |  | 0.751 |
| AA(C20:4n6) |  | Ref. |  | -0.02(-0.20, 0.16) | 0.829 |  | -0.08(-0.27, 0.11) | 0.396 |  | 0.393 |
| **Ratio** |  |  |  |  |  |  |  |  |  |  |
| Total n-6/n-3 |  | Ref. |  | 0.13(-0.05, 0.31) | 0.146 |  | 0.20(0.02, 0.39) | 0.033 |  | 0.031 |
| AA/EPA |  | Ref. |  | -0.04(-0.22, 0.15) | 0.690 |  | 0.03(-0.15, 0.21) | 0.763 |  | 0.754 |

^a^. model was adjusted for pregnancy factors and infant factors, which included maternal age, educational level, family income, gestational diabetes, pre-pregnancy body mass index, passive smoking during pregnancy, infant age and sex, feeding status at 6 months.

Abbreviations: PUFA: Polyunsaturated fatty acids; ALA: α-linoleic acid, EPA: eicosapentaenoic acid; DPA: docosapentaenoic acid; DHA: docosahexaenoic acid; LA: linoleic acid; GLA: γ-linolenic acid; α-linolenic acid; DGLA: dihomo-gamma-linolenic acid; AA: arachidonic acid; T3: tertile 3; T2: tertile 2; T1: tertile 1.
